# Smart Home Privacy Protection Methods against a Passive Wireless Snooping Side-Channel Attack

**DOI:** 10.3390/s22218564

**Published:** 2022-11-07

**Authors:** Mohammad Ali Nassiri Abrishamchi, Anazida Zainal, Fuad A. Ghaleb, Sultan Noman Qasem, Abdullah M. Albarrak

**Affiliations:** 1 School of Computing, Faculty of Engineering, Universiti Teknologi Malaysia, Johor Bahru 81310, Malaysia; 2 Computer Science Department, College of Computer and Information Sciences, Imam Mohammad Ibn Saud Islamic University (IMSIU), Riyadh 11432, Saudi Arabia

**Keywords:** smart home, data privacy, side-channel attacks, IoT security, wireless snooping attack

## Abstract

Smart home technologies have attracted more users in recent years due to significant advancements in their underlying enabler components, such as sensors, actuators, and processors, which are spreading in various domains and have become more affordable. However, these IoT-based solutions are prone to data leakage; this privacy issue has motivated researchers to seek a secure solution to overcome this challenge. In this regard, wireless signal eavesdropping is one of the most severe threats that enables attackers to obtain residents’ sensitive information. Even if the system encrypts all communications, some cyber attacks can still steal information by interpreting the contextual data related to the transmitted signals. For example, a “fingerprint and timing-based snooping (FATS)” attack is a side-channel attack (SCA) developed to infer in-home activities passively from a remote location near the targeted house. An SCA is a sort of cyber attack that extracts valuable information from smart systems without accessing the content of data packets. This paper reviews the SCAs associated with cyber–physical systems, focusing on the proposed solutions to protect the privacy of smart homes against FATS attacks in detail. Moreover, this work clarifies shortcomings and future opportunities by analyzing the existing gaps in the reviewed methods.

## 1. Introduction

### 1.1. Overview

The smart home is a concept that has existed for a few decades [1,2,3] and has promised technologically improved living environments to enhance the quality of residents’ domestic lives. In recent years, due to the emergence and rapid growth of the Internet of Things (IoT) and machine intelligence solutions, this technology has further evolved from a remotely controlled or automated home to a more realistic, smart home. Modern IoT-based smart homes provide various services to enhance convenience and residents’ control over their buildings while combining several intelligent devices. Some of the offered benefits of current smart homes include the management of and services for the following: temperature [4], ambient lighting [5], utility consumption [6], internal surveillance [7], safety risks such as fire incidents [8], physical security [9], intrusion incidents [10], and health monitoring for elderly people, kids, and pets [11]. However, taking advantage of the mentioned services is not entirely risk-free for users; e.g., there is a privacy violation risk.

IoT systems depend heavily on wireless communication solutions. Since a home network carries a remarkable volume of residents’ personal information, any data breach incident can be a catastrophe for users—for instance, the unauthorized disclosure of lifestyle, health conditions, political views, and financial situations [12,13]. In many cases, potential damages from a data leakage incident in a smart home would be complicated to compensate, if not impossible. Therefore, rising smart home privacy concerns have motivated researchers to seek appropriate solutions to mitigate this risk. In this work, we review threats from side-channel signal eavesdropping attacks briefly, then mainly focus on the FATS attack to investigate the latest attempts at and achievements in countering this problem, and the existing research gaps.

### 1.2. Background

Side-channel attacks (SCAs) exploit systems without having complete control over the devices; the attackers lack access to the source codes, communication contents, and detailed knowledge about system functionality. Encryption solutions cannot prevent these attacks, since the attack model is not interested in the content of the communications. Conversely, physical aspects, system responses, and contextual data related to a smart device’s wireless communication provide data-rich resources to infer critical information. 

A fingerprint and timing-based snooping (FATS) attack is an SCA that eavesdrops on emitted signals from a home perimeter and converts them into insights about the in-home activities of the residents. Passively violating the victims’ privacy puts them in a problematic situation. First, they cannot detect the attack during its operation; secondly, the leaked data can be used for more severe threats, e.g., blackmailing and selling data to a third party. To overcome the risk of FATS attacks, researchers have attempted to develop proactive defensive strategies for sabotaging the attack’s performance since it cannot be blocked or stopped. The foundation of this method of privacy protection is concealing the wireless traffic patterns of the actual events and obfuscating the correlations between the transmissions, either by adding a deliberate random delay before packet transmissions or by an injection of numerous fake packets identical to the actual ones into the network traffic.

The challenges in devising a privacy-preserving mechanism for smart homes against FATS attacks go beyond enhancing the privacy rate or diminishing the attack accuracy rate. A practical method must provide an optimal trade-off between the provided privacy rate, the system latency caused by the method, and energy demands. An investigation of the existing protection solutions indicates that many of these approaches’ drawbacks lie in their poor performances regarding energy consumption and system response time. Reporting actual events with a delay harms the quality of service, and random injections of fake packets drain the system’s energy resources.

### 1.3. Contribution and Scope

This article provides a novel overview of the existing protection solutions against “fingerprint and timing-based snooping (FATS)” attacks as passive side-channel attacks that effectively invade the data privacy of smart homes. The aim of this work is to elaborate on the latest solutions’ advantages and discuss their shortcomings to provide comprehensive insight on how to tackle the privacy-related vulnerability of smart homes.

### 1.4. Organization of the Paper

This paper consists of six sections. The first section covers a brief overview of the problem’s background and states the work’s contributions. Section 2 explains the security challenges related to smart homes by focusing on privacy issues. Section 3 describes the concept of the side-channel attack and its sub-categories associated with smart homes, concentrating on the details of FATS attacks. Section 4 reviews defensive strategies and existing privacy protection methods. Section 5 discusses the strengths and weaknesses of the reviewed solutions. Finally, Section 6 concludes the paper by providing recommendations for future research directions.

## 2. Smart Home Security

### 2.1. Smart Home Overview

**Figure 1:**
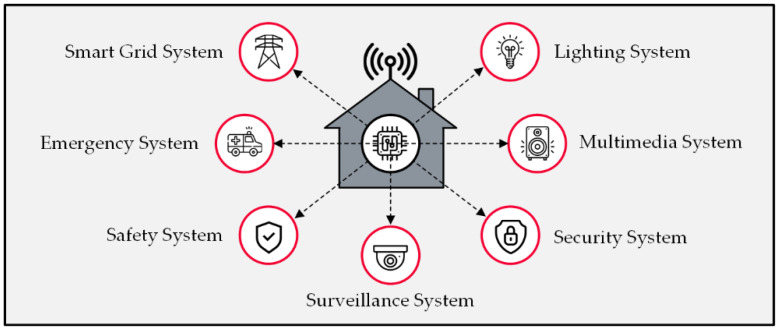
Smart home applications.

A smart home is a residential building with several interconnected smart systems; these embedded systems provide some advanced digital services to users, such as remote healthcare monitoring, the intelligent management of utilities, high-level security surveillance, etc. [14,15]. The rapid evolution of enabler technologies, such as IoT, AI, and wireless communication solutions, promotes the smart home’s attractiveness to users. IoT sensors and actuators are being developed for various applications while becoming more cost-effective [16,17]. Moreover, improved wireless protocols offer lightweight solutions with a broader signal coverage, better connection stability and reliability, lower energy demand, and more robust security measures [18,19]. Machine learning also contributes to developing numerous solutions, such as chatbots [20], automated video analyzers for CCTVs [21], anomaly detection solutions [22], and intelligent computer–human interaction technologies [23]. Despite the beneficial applications of a smart home, these systems raise concerns, mainly regarding information security and privacy, since they are prone to data leakage, which might result in catastrophic consequences for users [24,25]. Figure 1 illustrates a schematic view of a smart home and a few of its applications.

### 2.2. Smart Home Privacy

Privacy-related threats to smart homes are among the most significant risks that need to be addressed. However, an extensive list of cyber attacks might compromise the functionality of smart home systems. Ensuring the security of the personal information of smart home residents is a vital requirement to eliminate the threats associated with the wide acceptance of such systems [25]. The data transmitted over an IoT wireless network are divided into system data and users’ data. These categories of data distinguish the required security measures. One needs data confidentiality, and the other demands data privacy.

#### 2.2.1. Data Confidentiality

Confidentiality in a wireless system refers to properly concealing the contents of data packets, which include either controlling messages or information about the functionality of smart devices, and preventing unauthorized access by intruders [21]. Implementing cryptographic methods is a common way to protect these messages within an IoT system. The complexity of the advanced encryption methods challenges attackers to find the secret keys to reveal the plaintexts and ensures that the system’s information is not discoverable by those who are not permitted [22]. On the other hand, the drawback of encryption techniques is that they leave the contextual data of network messages unprotected. Examples of these sorts of data are the smart device’s identity, location, and activity time. This type of data provides a rich resource for attackers to obtain critical information about the system, which may be worth more than the contents.

#### 2.2.2. Data Privacy

Data privacy indicates that the protected information belongs to a person rather than a device or system. Residents of an intelligent building share extensive data about their matters with the system, and numerous embedded sensors in various smart devices are in charge of collecting data about users’ Activities of Daily Living (ADL). These comprehensive accumulated data enable the system’s intelligent engines to evaluate the situation of users and make services to comply with their desired needs. Likewise, the system’s ability to acquire data provides a valuable resource for intruders to discover sensitive information about residents [26].

Based on the definition, privacy means the right someone has to keep their personal life or personal information secret or known only to a small group of people [27], which is protected by law in most countries. The motivations of hackers to violate their victims’ privacy rights vary from commercial benefits to personal hostilities [28,29]. In some cases, governments violate citizens’ privacy through illegal surveillance [30,31]. 

**Figure 2:**
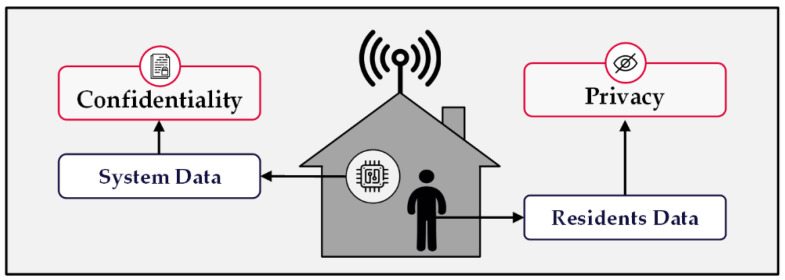
Types of home data and their related security measures.

In the case of FATS attacks, intrusion detection systems do not help due to the passive nature of the attack. The attacker quietly collects the data and abuses them; the catastrophic results of the snooping appear once it is too late for the victims to properly react. Moreover, encryption methods fail to secure the intended information by the attackers since the data leak from the contextual aspects of the wireless transmissions. Therefore, the above situations emphasize the need for a robust, proactive defense mechanism within home systems to protect users’ information. Figure 2 demonstrates the difference between confidentiality and privacy in terms of data type.

## 3. Side-Channel Attacks

Conducting a side-channel attack means exploiting the physical aspects of a device to discover its associated critical information. The primary assumption in SCAs is that data are constantly leaking; therefore, attackers have opportunities to take malicious advantages of the system [29].

SCAs are divided into active or passive attacks. Active SCAs require physical access to the targeted device/system or physical proximity. An example of an active SCA is the fault analysis attack, in which hackers inject predefined inputs and observe the system’s response; through this process, they intend to discover how the device works. Another example is analyzing the sounds produced by a device and discovering their correlations with the system’s functionality, which is more applicable to systems with mechanical actuators. A potential countermeasure to these attacks is physical security solutions. In contrast, passive SCAs are not recognizable by the victims during the attack. They quietly exploit the external aspects of a device. For example, an eavesdropping attack remotely listens to the network traffic and captures transmitted data packets for further analysis. Even though the captured packets have been encrypted, attackers can extract valuable information from the contextual aspects of the signals carrying those data packets. A suitable solution to overcome the risk of passive SCA attacks is to employ proactive defensive methods to prevent the attackers from interpreting the stolen packets [7].

### 3.1. Vulnerabilities of Smart Systems

A typical smart device developed for a smart home usually has several standard building blocks, such as a processor, I/O ports, data storage disk, wireless communications modules (Wi-Fi, Bluetooth, etc.), power supply unit, and, depending on the device’s application, a group of sensors and actuators. For attackers who utilize SCAs, each device component is prone to exposing valuable data. Therefore, in the last decades, numerous malicious techniques have been developed for invading various aspects of intelligent devices [32,33,34]. 

**Figure 3:**
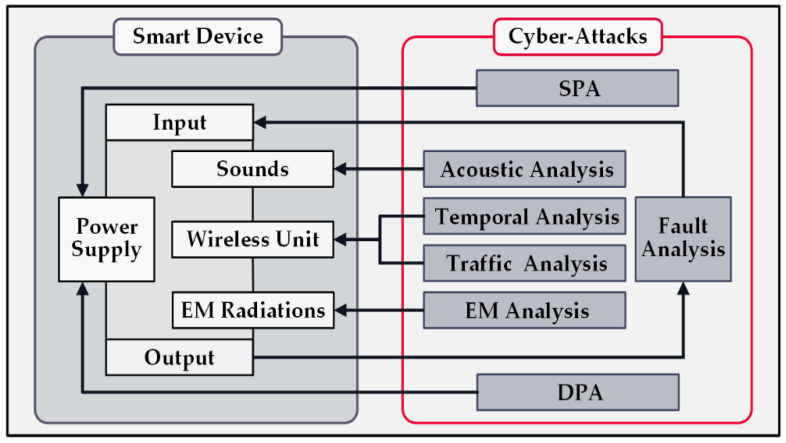
Side-channel attacks on a smart device.

As illustrated in Figure 3, each embedded unit in the device faces at least a corresponding attack. A simple power analysis (SPA) and differential power analysis (DPA) are two attacks that threaten the system through the power supply unit. A system’s wireless communications are the targets of temporal and traffic analyses. Moreover, a fault analysis attack exploits the I/O ports of the device. In addition, acoustic and electromagnetic analyses extract private information from the emitted sounds and EM radiations of the device.

### 3.2. Side-Channel Attacks Categories

**Figure 4:**
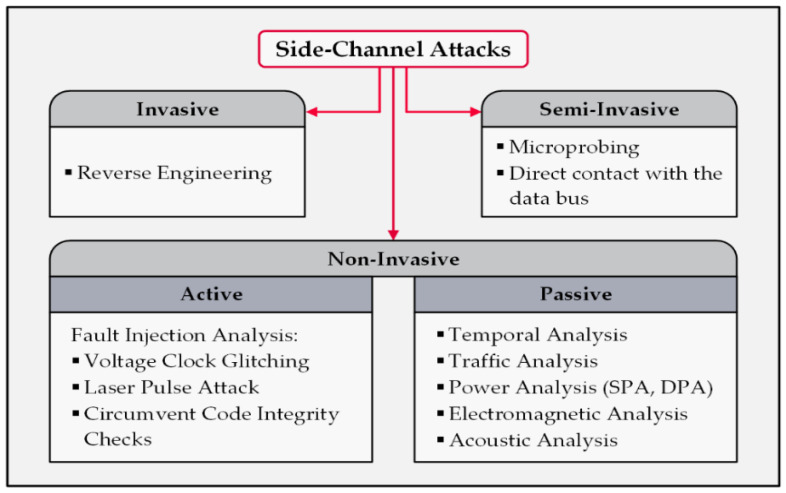
Taxonomy of the side-channel attacks on cyber–physical systems.

Side-channel attacks are divided into three classes from a physical security perspective: invasive, semi-invasive, and non-invasive. An invasive attack physically manipulates the targeted device which destroys the device most of the time. Damaging the components to study their functions and conduct chemical examinations are examples of this class of SCAs. Similarly, semi-invasive SCAs need physical manipulations but do not dismantle the devices—for instance, opening the enclosure to gain direct access to the PCB or disassembling some parts. In contrast, non-invasive SCAs only exploit accessible information via data ports, external power cables, wireless communications, emitted electromagnetic radiations, or produced sounds. These items provide valuable contextual data about the system and its functionality [35]. Figure 4 demonstrates the taxonomy of SCAs.

- A simple power analysis (SPA) monitors the alterations of a device’s consumption of power when executing various algorithms and reveals a series of patterns that can be correlated to specific activities. Attackers attempt to match these extracted patterns with known algorithms to identify the device’s function. For example, this method can differentiate between encryption methods since their energy demands are different. One might note that occasional current spikes and noises are challenges that limit the performance of this approach [36].
- A differential power analysis (DPA) is an improved power analysis method that applies statistical error-correcting approaches. This method monitors the power consumption of a device to discover the encryption key. The primary difference between the DPA and SPA methods is that the DPA method analyses the device’s power consumption for two types of operations, non-cryptographic and cryptographic; then, it compares the results to discover the system’s critical information. A DPA is a powerful tool that threatens all sorts of hardware protected by cryptographic approaches [37,38].
- A fault analysis is an approach in which the attacker injects various types of faulty inputs into a smart device and then investigates the outcome of the system. A few examples of physical hardware manipulations are increasing the device’s temperature, applying a laser beam at a specific frequency, and injecting fake inputs to surge the likelihood of the signals’ collision [39,40].
- An electromagnetic analysis takes advantage of the emitted power radiations from devices protected by encryption approaches while performing encryption and decryption processes to discover the correlation between EM radiations and the ciphertext. This attack does not require proximity of the attacker system to the target device, depending on the radiation receiver equipment’s strength, which makes it suitable for remote performance [41].
- An acoustic analysis takes the produced sounds (noises) by the electromechanical components of a device as the input and attempts to obtain the system’s secret information via analyzing the acoustic oscillations. The advantages of this attack are the capability of the attack algorithm to precisely distinguish the slightly different sounds and its use of relatively simple equipment, such as a digital sound recording device or a smartphone [42,43,44].
- A timing analysis concentrates on temporal data related to the wireless signal transmissions of a system. The attack aims to discover the temporal correlations in the network traffic and recognize the patterns, which reveals critical information about the system’s behavior. This malicious approach is a fitting option for an eavesdropping attacker who has a global view over the system’s communications, especially if they are interested in a system’s contextual information [45].
- A traffic analysis includes a wide range of options for scrutinizing the system’s network traffic. This approach mostly focuses on transmitted data packets over the wireless network. The number of packets, the packets’ associated signal fingerprints, and the correlation between the packets forwarded from different devices are valuable contextual data for an attacker in identifying the sender, receiver, and their locations [46].

## 4. Data Snooping Attack from Smart Home

Due to their passive nature, eavesdropping-based cyber attacks are among the most challenging threats for cyber–physical systems. However, several cryptographic solutions have been developed to secure the content of communications. A sub-class of these attacks aims at the contextual data of the system; therefore, encryption methods would be irrelevant to ensuring system data security while encountering such threats. In this paper, we investigate an attack of this sort called a fingerprint and timing-based snooping (FATS) attack [47]. This attack begins with eavesdropping on the signals emitted from a smart home from a nearby location within the coverage of the home’s wireless network (e.g., a neighbor’s house), even when all the data packets are encrypted. Then the attack algorithm analyses the captured data packets based on their signal fingerprints and timestamps to discover the victims’ activities of daily living. This situation is a clear example of a privacy violation.

The stolen data from a home network may contain sensitive information about the residents’ lifestyle, political views, financial situation, personal health conditions, sexual conduct, routines, future schedules, shopping preferences, and more. Therefore, an attacker would be capable of launching subsequent severe attacks to harm their victims using this information. Notable examples of probable risks include blackmailing, selling information to a third party, terrorism, defamation, and government surveillance.

Implementing FATS attacks consists of multi-tiers in which a combination of machine-learning techniques, such as classification, clustering, and features-based matching, takes place. The attack clusters the captured signals based on their radio fingerprints and attempts to find correlations between data packets’ forwarding timestamps. Eventually, the algorithm identifies the connected devices, rooms, and in-home events; from this point onwards, the hacker obtains unauthorized monitoring access to the smart home. The four tiers of this attack are as follows:

- Tier 0 detects smart devices based on their unique radio signatures. The fingerprint of data transmission is a set of RF waveform features that differentiate the source of signals even if the signal senders have a similar manufacturer and model. The attack identifies basic events, such as home occupancy or sleeping, in this step.
- Tier 1 clusters the identified nodes by investigating time intervals between signal transmissions. The assumption is that devices which are spatially located close to each other activate at a proximate time. Thus, the produced clusters represent either the location of their member devices, e.g., a room, or their purposes, e.g., cooking.
- Tier 2 performs clustering labelling using the extracted features from the formed clusters. Note that this process prioritizes logical categorization rather than device locations. For example, the washing machine will be categorized as a laundry event, although it might be located in the kitchen or basement.
- Tier 3 performs another round of classification in which the attack algorithm uses the extracted feature vectors of the node clusters and the training data to label those clusters as the devices. For example, the attack’s trained model expects the stove to be in the kitchen; therefore, it examines the clusters placed in the kitchen cluster with the stove’s features vector extracted from the actual dataset. In the case of a proper match, the unknown device cluster would be recognized as the stove.

**Figure 5:**
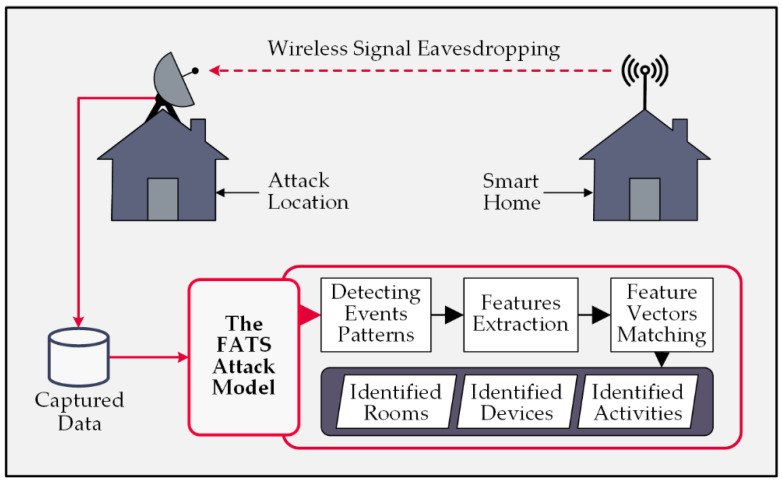
Overview of the FATS attack processes.

The FATS attack procedure combines several statistical and machine-learning techniques to discover the identities of rooms, devices, and activities. First, it clusters the records based on the radio fingerprints. Then, using the associated timestamps, it creates a temporal matrix representing the time proximity between the transmissions. Next, it converts the temporal matrix to a metric distance matrix using Dijkstra’s shortest path algorithm [48]. It applies classical non-parametric multi-dimensional scaling (CMDS) to produce a position matrix based on the distance matrix [49]. Finally, the K-mean clustering algorithm [50] clusters the device clusters that are temporally correlated. Figure 5 shows the operational diagram of FATS attacks.

**Figure 6:**
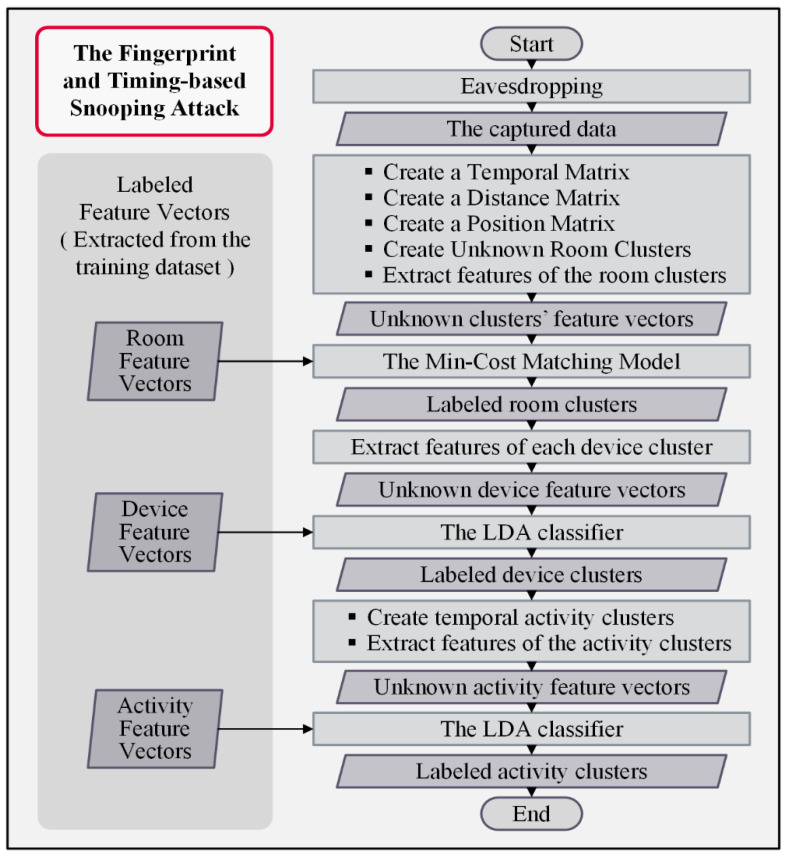
Flowchart of FATS attacks.

The attack converts the inputs to the devices’ geospatial information. Then, it attempts to identify devices, rooms, and activities by matching the produced feature vectors from the created unknown clusters to the known vectors for its model using some classifier models. As a result, it successfully violates the residents’ privacy without cracking the secret key of the encryption. Figure 6 provides further details on the inside processes of FATS attacks.

The attack labels the room clusters in the next tier by computing the maximal min-cost bipartite mapping [51]; the critical features for the matching process are the number of transmissions per day from the room, the total number of transmissions during the day and at night, the median inter-transmission time within a room, and the median length of temporal activity clusters. In the subsequent process, the attack identifies the devices by extracting features of devices and then matching them with the known feature vectors for the trained model; a standard linear discriminant analysis (LDA) classifier [52] performs this task. Afterwards, creating temporal activity clusters in every device cluster results in several activity feature vectors. These features are the start time, duration, and the number of transmissions by each device. For the second time, the LDA classifier performs the matching task on the unknown activity feature vectors to relate them to a labelled vector of the attack model. Ultimately, the identities of all rooms, devices, and activities are revealed to the attacker; then, they can monitor all in-home activities and obtain the residents’ private information [47].

## 5. Privacy Protection Strategies against the FATS Attack

Investigations into the proposed methods developed to protect smart homes encountering FATS attacks indicate that researchers have attempted different defensive strategies, and in some cases, they have succeeded in remarkably improving the privacy rate. Although preserving privacy is the primary goal for every proposed solution, the implications of the employed approaches on the other system parameters are critically important. For example, solutions that impose latency on the system communications decrease its quality of service; this side effect is especially unacceptable for a few delay-sensitive home systems, such as systems for the detection of fire and elderly people falling. In addition, the methods’ energy demands have to be justifiable. A suitable solution must have an optimal balance between the three system parameters, namely the privacy rate, communications latency, and energy consumption.

Confusing the pattern recognition mechanism in FATS attacks is the main idea of the defensive strategies to mitigate the risk of data breaches; for this purpose, the preferred approach is to obfuscate the signal traffic of the smart home’s wireless network. Since the attack algorithm’s foundation is finding the temporal correlations between transmitted signals, manipulating the actual patterns sabotages the attack’s performance.

Strategies for developing a protection method fall into two categories. In the first category, solutions delay sending the data packets. The delay durations are randomly determined; therefore, the attack would have difficulty finding the actual time correlations between the transmitted signals. On the other hand, methods in the second category randomly inject some fake packets into the network traffic, and the characteristics of these dummy packets are identical to the actual ones; therefore, the attack cannot differentiate between them. As a result, the accuracy of the FATS attack diminishes because it will be challenging for the attacker to properly understand the actual events occurring in the home.

### 5.1. Late Packet Injections Technique

**Figure 7:**
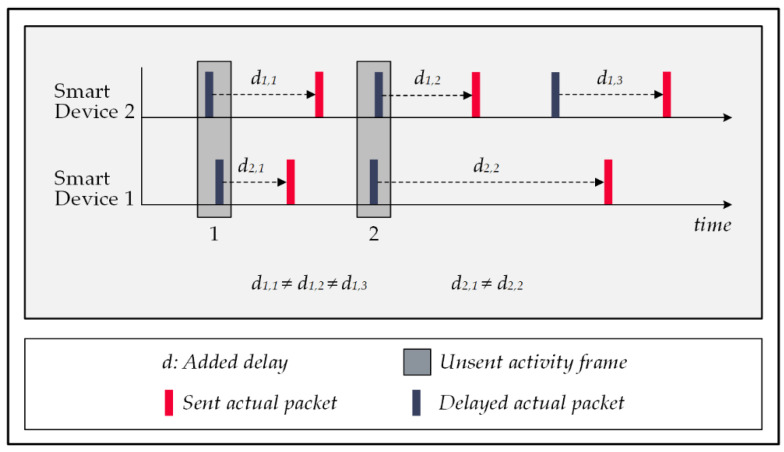
Temporal manipulation in forwarding actual data packets.

The temporal manipulation of signal traffic refers to altering the actual time correlations of signals transmitted by devices that collaboratively report an event. To do this, devices use a random interval generator algorithm to delay their packet transmissions for a random amount of time. The primary assumption of the attack is that devices collaborating in reporting an event send out their messages at a proximate time to each other; therefore, the deliberate change in transmission times prevents the attacker from finding the real activity patterns, resulting in a decline in the attack’s accuracy. The drawback of this strategy stems from the excessive latency imposed on the system’s communications. Some of the smart home’s sub-systems are delay-sensitive, e.g., healthcare-monitoring systems, because delayed communications affect their response time, a critical factor in their performance. Figure 7 shows a sample signal traffic manipulation for the delayed reporting strategy, in which activity patterns 1 and 2 are scattered over the time to prevent the correlated signals from being framed as activity by the attack’s event-pattern-detection mechanism. It is important to note that the data packets are encrypted and include information that lets the central controller figure out how they really relate to each other [53].

### 5.2. Fake Packet Injections Technique

**Figure 8:**
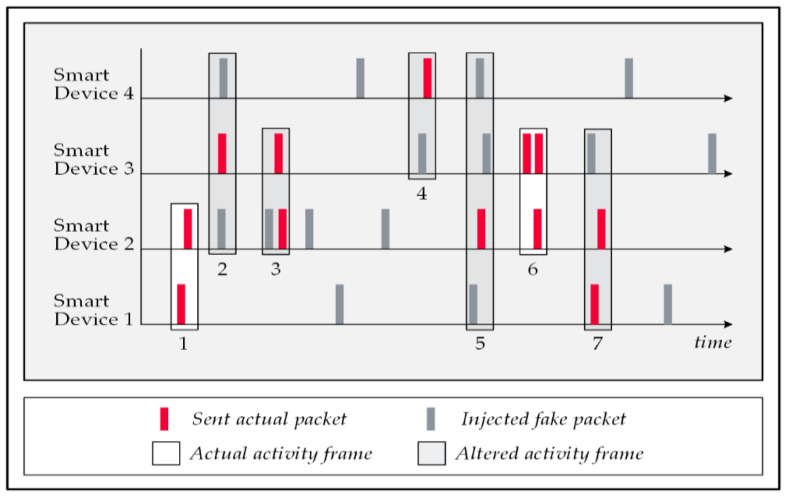
Fake data packet injections.

Injecting a series of dummy data packets into the home’s wireless traffic is an alternative approach to mislead the FATS attack. In this strategy, the system tasks the smart devices to generate a random number of data packets identical to actual ones and transmit them in random intervals. The forged messages are encrypted; therefore, the attack cannot distinguish them from the actual packets; therefore, it considers them in its pattern recognition processes and this mistake leads the attack algorithm to make false conclusions. The falsehood of the results is either due to the attack’s failure to detect the patterns of actual events (false negative cases) or to reporting some activities that never occurred (false positive cases). Both of these mistakes affect the correctness rate of the attack. Figure 8 illustrates a schematic view of the network traffic in which the attack has formed six activity frames; frames 1 and 5 are correct detections, frame 6 is a false positive, and frames 2 and 3 are undetected events by the attack [54,55,56].

### 5.3. Hybrid Techniques

**Figure 9:**
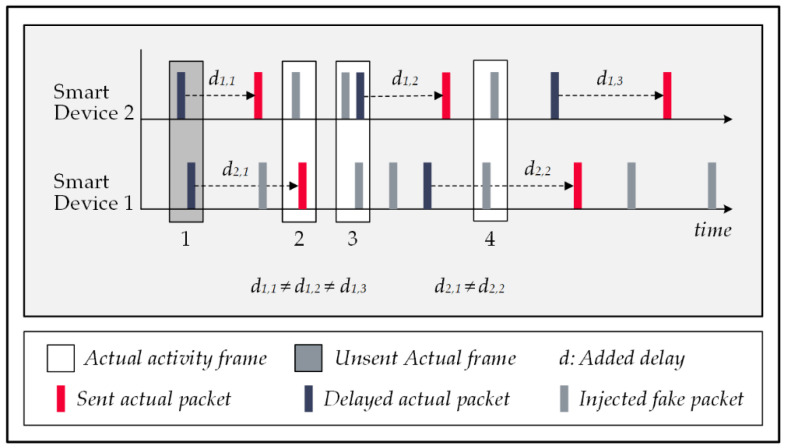
A hybrid of temporal manipulation and fake packet injection techniques.

As shown in Figure 9, combining the previous defensive strategies in a method brings their protection benefits together. In the demonstrated traffic example, frame 1 relates to an actual event, but the attack cannot detect it since the timing of the forwarding of data packets has shifted. On the other hand, the attack recognizes frames 2, 3, and 4 as probable actual activities that, in all of them, had injected dummy packets that deceived the attack; therefore, none of them would be matchable with any known activities by the trained model [55,57].

## 6. Related Works 

Data privacy and security in wireless network systems (WSN) and the Internet of Things (IoT) have been studied extensively in recent years. In this domain, several research studies have focused on the sub-category of smart buildings and attempt to address concerns such as source anonymity [58], data eavesdropping [59], and false data injection [60] attacks. This section reviews privacy-preserving methods for smart homes encountering FATS attacks; these proposed approaches attempt to mitigate the risk of passive wireless snooping cyber threats by obscuring the traffic patterns of in-home daily activities. 

In [53], the authors argued that every smart device must define a series of injection windows with a constant interval and forward all data packets within these periods. The proposed method is called the ConstRate scheme. In this method, the device postpones the reports related to detected events until the upcoming injection window. The system forges a dummy message to fill the injection period if no actual packet exists. 

An argument for the effectiveness of this scheme is that establishing a transmission framework from the beginning of the process results in a uniform distribution of signals in the network traffic; therefore, finding any time correlations in the transmitted signals is nearly impossible for the attack. Failing to recognize actual traffic patterns disarms the FATS attack completely. Simulation results of the ConstRate scheme support this claim by showing a near-perfect privacy protection rate. 

**Figure 10:**
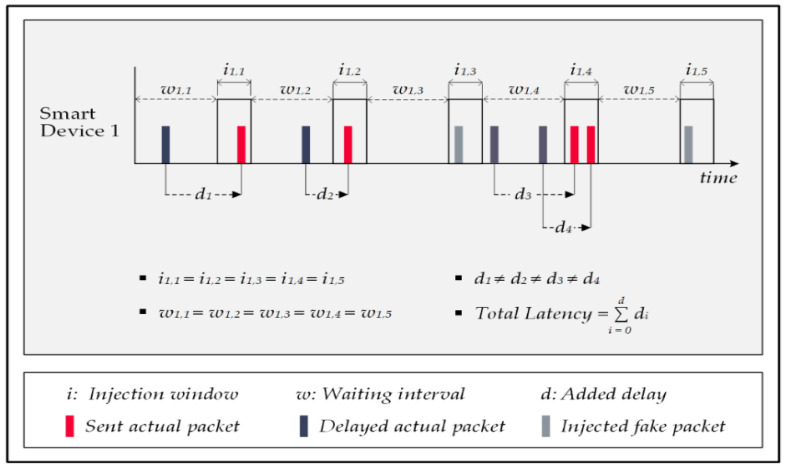
The ConstRate scheme.

On the contrary, the drawback of the ConstRate scheme is its detrimental effect on the system’s response time and energy efficiency. The determination of the waiting interval is random in this approach. Thus, on the one hand, the shorter waiting intervals enforce a higher number of fake packet injections and surge the system’s energy demand. However, on the other hand, the longer intervals increase the transmission delays for the actual messages, which prolongs the overall latency within the system. Neither of these consequences comply with the required optimal trade-off for the critical factors of the system. Figure 10 demonstrates the principles of the ConstRate scheme with sample traffic. All injection windows have equal durations, and the waiting intervals are similar.

In response to the shortcomings of the ConstRate scheme, researchers proposed the ProbRate scheme in [53]. In this approach, an exponential distribution determines the waiting intervals, meaning every random interval must be on the designated distribution; otherwise, the system discards it and then repeats the process. 

**Figure 11:**
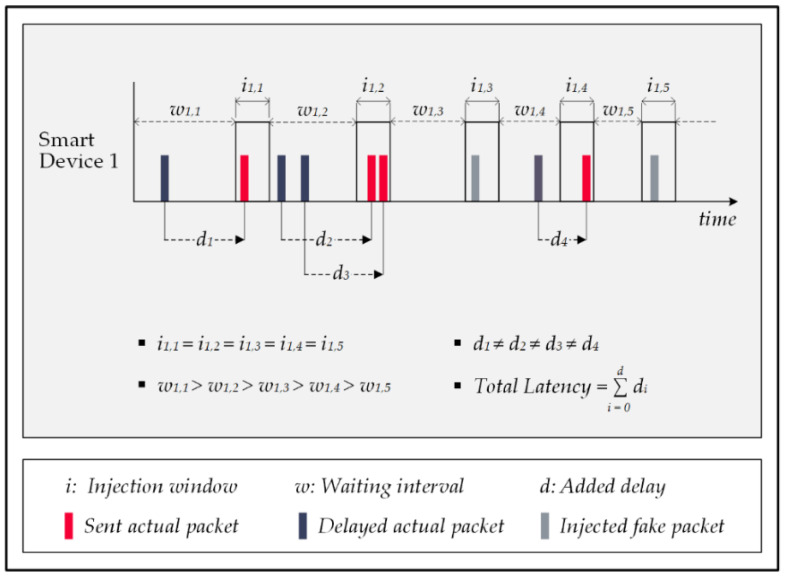
The ProbRate scheme.

The chosen intervals get shorter over time by following an exponential distribution, producing shorter packet-forwarding delays, resulting in less overall latency in the system. This method keeps the smart home’s privacy rate high despite the attack’s capability of viewing all the traffic, recognizing the distribution pattern, and obtaining its mean over time. Observing a repetitive pattern in the network traffic decreases the attack’s capacity to identify the needed time correlations, and similar to the previous scheme, the ProbeRate scheme offers near-perfect privacy protection for smart homes against FATS attacks. Figure 11 illustrates how the ProbRate scheme manipulates the network traffic to conceal the actual patterns.

Although the ProbRate scheme reduces delays, it does not eliminate the overall system latency. The fact is that this issue is still problematic for delay-sensitive systems in smart homes. Additionally, by reducing the waiting times for the packet injections, the number of injection windows increases, which increases the likelihood of injecting fake messages; thus, the systems’ energy consumption escalates.

The FitProbRate (FPR) scheme is another approach for preserving privacy in a smart home facing FATS attacks as proposed in [43]. This scheme is an upgraded version of the ProbRate scheme; thus, the system determines the waiting intervals based on an exponential distribution, similar to the previous method. Moreover, the FPR scheme employs the Anderson–Darling test [61] to ensure that every chosen interval belongs to the value set of the exponential distribution. Moreover, this approach controls the deviation between the measured sample means and the actual mean of the designated distribution to avoid a significant difference between them. In addition, the scheme prioritizes forwarding the actual packets as soon as possible; therefore, the system sends out the data packet after the shortest waiting time that fits into the given distribution and reschedules the injection of the prepared fake packet to the subsequent injection window. As a result of the employed strategy, the waiting intervals shrink gradually, and the injection windows come closer to each other, reducing the overall delay. 

**Figure 12:**
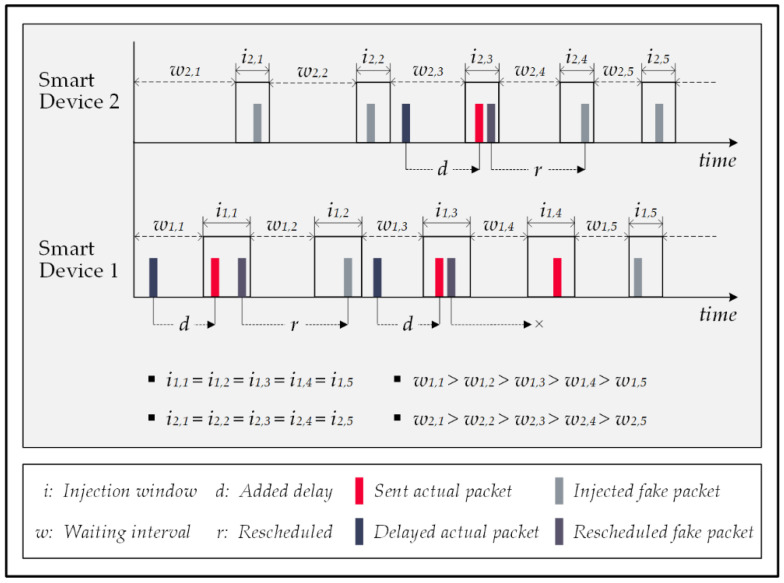
The FitProbRate scheme.

According to the authors’ reported results, the FitProbRate scheme’s system latency is approximately one-tenth that of the ProbRate scheme, which indicates a remarkable improvement in terms of maintaining the quality of service of the smart home. Conversely, the proposed scheme does not offer any improvements regarding the energy overhead issue. In this scheme, smart devices must inject at least a dummy packet in every injection window that is empty of actual messages to disrupt the performance of the attack’s pattern recognition algorithm. As a negative effect, for a long period of silence in the home, e.g., at night, the system consumes a massive amount of energy for injecting unnecessary fake packets since there are no actual patterns to conceal. Moreover, similar to earlier schemes, the number of injected fake packets is an uncontrollable random value which undesirably affects the energy demand of the scheme. Figure 12 shows a sample of traffic pattern manipulation by the FitProbRate scheme. In this example, an actual packet has shifted from the third waiting time to the third injection window, and the system has rescheduled the injection of the pre-planned fake packet for this window to the fourth injection slot.

In a subsequent work [55], the authors proposed a novel concept compared to the prior schemes, which were founded primarily on utilizing statistical distributions. In this method, the protection mechanism analyzes the behavioral semantics of home events and trains its decision-making model with the home’s historical records. The main aim is to predict the likelihood of the occurrence of actual events; thus, the system can purposefully inject fake packets to interfere with the actual signals traffic, altering the activity patterns and decreasing the FATS attack’s event-detection accuracy. Although this method reduces the system’s energy overhead, it fails in addressing the added latency issue since sending the data packets must happen in the predefined injection windows. Furthermore, the method’s success is highly dependent on the correctness of its predictions. Wrong forecasting wastes the consumed energy on transmitting dummy messages.

**Figure 13:**
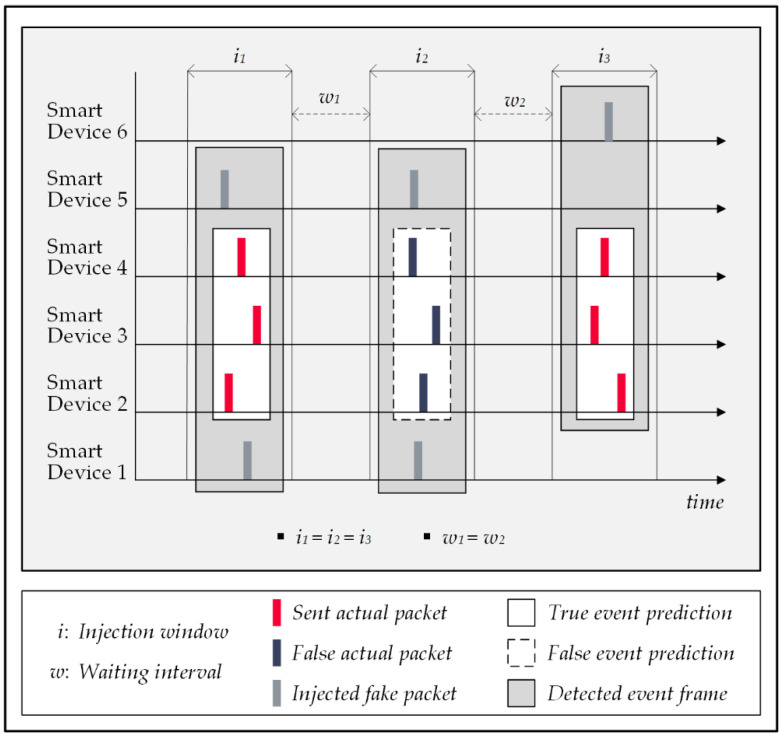
Privacy-preserving method based on events’ behavioral semantics.

Figure 13 shows a traffic pattern protected by the events’ behavioral semantics method for preserving the privacy of the home’s information. However, the FATS attack has detected three activity frames. It cannot identify any activities since the detected frames are not matchable with any of the attack’s known activity patterns. In the first and third injection windows, the protection method has correctly predicted the events’ occurrence; thus, the interferences of the injected dummy packets by other devices have made the traffic patterns unrecognizable for the attack. The event prediction for the second injection window has been incorrect; as a result, the injected fake packets have not contributed to concealing the in-home activities, and the consumed energy has been wasted.

The latency issue of the privacy-protection method is significantly reduced in [57], in which the authors proposed a real-time adaptive approach using supervised learning techniques to cope with the risk of FATS attacks; this method is called the sample data and supervised learning (SDASL) method. The method is claimed to have a low latency, low energy consumption, strong adaptability, and adequate privacy protection for smart homes. In the SDASL procedure, the central controller computes decision parameters periodically for every smart device and then applies a logistic regression algorithm to determine whether the device must send out a fake data packet. The latest network traffic state is a critical factor in the decision-making process for this method. 

The SDASL consists of two phases, sample data analysis and supervised learning. In the first step, the model simulates the dissemination of fake messages using the distribution of the radio frequencies (RF); the output is referred to as the FDR. Firstly, the similarity in the extracted frequency rates from the sample dataset and dummy messages is critical. Then the central controller updates the smart devices with the produced FDR. In the second phase, a supervised learning model is employed to perform three tasks: data collection, labelling, and learning model parameter updating. Every device in the home network must be upgraded with a copy of the final prediction model; the required inputs for this model are time and the network’s traffic status. A logistic regression function makes the final decision using the real-time inputs and a threshold set of 0.5; computation results over the passing mark indicate that the fake packet must be injected. The SDASL method requires frequent communication between the central controller and the smart devices. 

Reportedly, the SDASL method decreases the FATS attack’s accuracy by 30% after 13 days of model training, which means a 70% privacy rate for the home. The achieved privacy protection is significantly lower than that of the aforementioned statistical-based schemes, but it resolves the need for injection delays. Energy-wise, the results state that for every actual data packet, 13 fake packets were injected, meaning the energy cost of the SDASL method is thirteen times more than that of an unprotected home.

**Figure 14:**
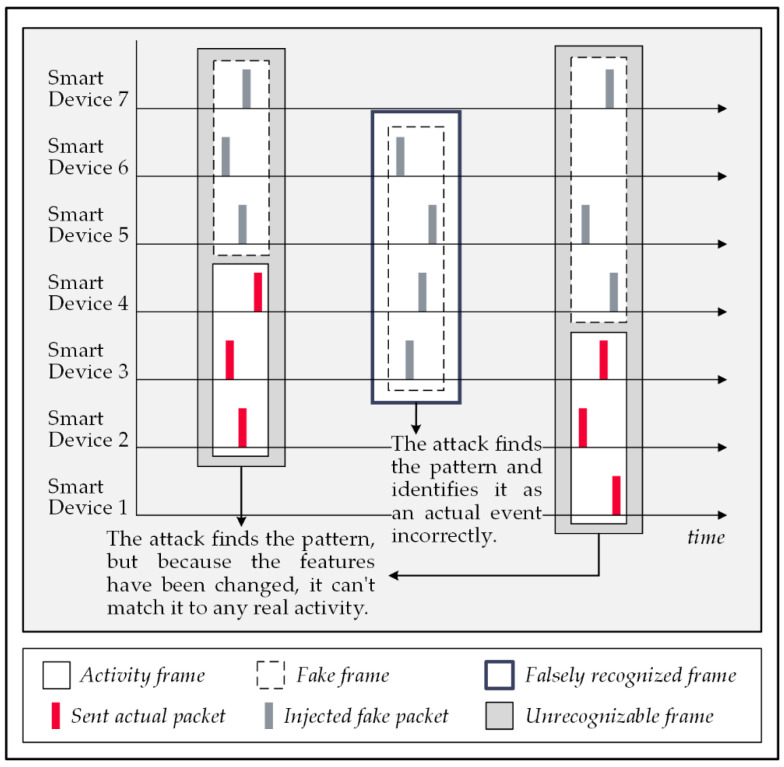
Actual activity mimicking method.

The authors in [56] briefly introduced a novel concept to counter FATS attacks and suggested a paradigm-shifting strategy for injecting fake packets. The proposed notion is to inject dummy packets collaboratively to impersonate an actual in-home activity; thus, even though these random injections do not interfere with an actual pattern, they can deceive the attack by themselves and prevent energy wastage. Figure 14 shows an overview of the impact of this method on the network traffic and performance of the attack. 

The reporting of fake events by the attack increases its true positive rate (TPR); therefore, the attack’s accuracy rate decreases effectively. This approach is called the actual activity mimicking (AAM) method and provides the most optimal trade-off for the triad of privacy protection, communication latency, and energy consumption. 

In this solution, the policy of immediately forwarding the actual data packet eliminates the latency issue. Moreover, it improves the privacy rate by deceiving the FATS attack to report unreal events, which decreases the TPR of the attack model. In addition, despite the randomness of the injections, this method increases the overlapping chance for fake and actual activity patterns, using a probabilistic-based mechanism that concentrates the injections on periods of the day with a higher likelihood of actual activities. This strategy enhances the privacy rate, consumes energy resources more efficiently, and complies with the zero-delay requirement for quality-of-service matters.

## 7. Discussion

Preserving the privacy of smart homes is a critical requirement that should be fulfilled to prevent unintended consequences of potential data breaches. FATS attacks effectively let attackers discover smart home residents’ private matters related to their in-home activities. The essential characteristics of this attack can be summarized as follows:

- This attack performs passively; therefore, it is undetectable during the attack period.
- The attack extracts information from contextual data of the home’s communications; thus, encryption methods cannot resist it.
- This malicious algorithm requires a minimum of inputs including the signals’ fingerprints and timestamps of the transmissions; hence, blocking its access to these data is challenging.

All things considered, since the attack is unstoppable, it is evident that mitigating the risk of this threat would be a proactive protection solution to ensure the traffic patterns of the snooped data are altered in a way that the malicious algorithm cannot interpret them precisely.

Investigating the existing protection methods indicates that the predominant tactic in these approaches is to maximize the obfuscation of the network traffic to challenge the attack’s pattern-recognition ability. It seems reasonable to assume that the poor performance of the attack in detecting activity patterns causes problems in subsequent phases. This aim is obtained by techniques such as the temporal manipulation of the transmitted signals or injections of random fake data packets, both effectively altering the traffic’s patterns. Nonetheless, this achievement comes with a cost for the system which appears as either latency in communications or an energy overhead. 

Notably, the home privacy rate has a reverse relationship with the attack’s accuracy; therefore, any decline in the attack’s correctness rate means an equal gain for the privacy rate. The two primary metrics to compute the attack’s correctness rate are the event detection rate (EDR) and the true positive rate (TPR) [47]. The EDR refers to the proportion of correctly detected in-home events from all actual activities. For example, identifying 75 actual events in a home in which 100 activities occurred produces an EDR of 75%. Moreover, the TPR indicates the report’s correctness percentage. For instance, in a result list containing 100 labeled events, if 60 items are false, the TPR would be 40%. Finally, the attack accuracy is the multiplication product of the EDR and TPR. Thus, the attack correctness rate using the above examples is 33.75%.

This study’s investigations have shown that most reviewed works emphasize decreasing the attack’s EDR as their primary aim. The logic behind this choice seems to be simple but effective; if the attack does not detect the events, it cannot identify them. The results of a few reviewed protection schemes support this argument, such as the ConstRate, ProbeRate, and FitProbRate schemes that provide near-perfect privacy for home systems. However, overlooking the drawbacks of these solutions would be costly. These approaches alter the time correlations of the transmitted signals by delaying the forwarding of them, which means there is an unbearable latency in the real-time services of the home. This issue affects the response time of delay-sensitive systems and harms their effectiveness. Examples include incidents such as an elderly person falling, the detection of a fire which requires an immediate report, or in a more straightforward case, the late performance of smart locks which can inconvenience users.

Moreover, concealing the actual activities and traffic patterns demands the injection of numerous fake packets; the number of these injections is not deterministic to comply with the necessity of randomness in the protection procedures. The results have shown that to provide an adequate privacy rate, the number of fake packets is many times more than that of the actual packets. In this regard, the notation of FVR refers to the ratio of the dummy packets to the actual ones. Since transmitting both types of data packets equally consume energy resources, the system’s energy overhead can be massive, which undermines the affordability of the solutions.

Unlike other methods, the actual activity mimicking method targets the TPR to decrease the attack’s accuracy. Unlike others that attempt to conceal the traffic patterns of the actual events, this method encourages the attack to detect activities as much as possible. Therefore, this matter provides the opportunity to blend more fake activity patterns into the final outcome of the attack which diminishes the TPR. Injecting fewer fake packets lowers the system’s energy consumption and eliminates delays in wireless communications, which are the advantages of this method over the other solutions.

**Figure 15:**
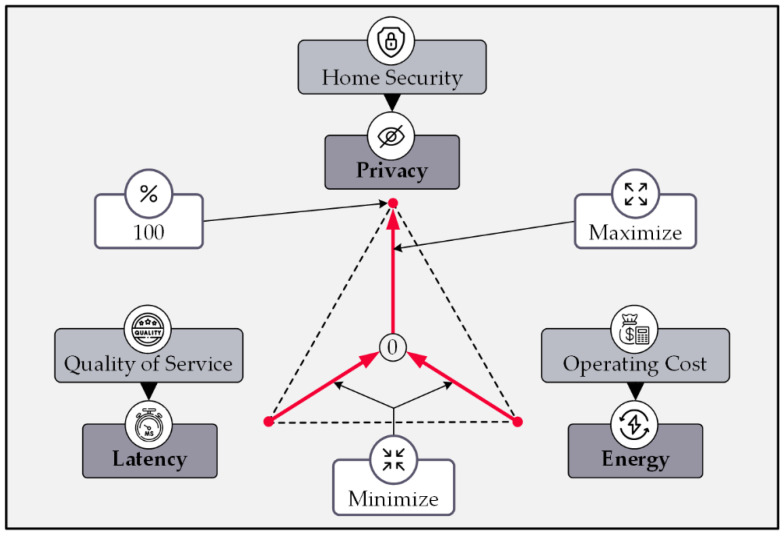
Triad of evaluation metrics for protection methods.

In sum, the critical metric for evaluating a privacy protection method is the circumstances of the trade-off between the provided privacy rate, the latency caused, and the energy consumption of the solution. A method will be ideal if it maximizes the privacy rate while keeping the two other factors as low as possible. Figure 15 illustrates the relationship between the parameters of privacy, latency, and energy mentioned above.

The first reason for the necessity of considering this trade-off is the existence of delay-sensitive smart home sub-systems and the need to comply with quality-of-service requirements. The second justification stems from the unintended increase in operating costs caused by the massive energy overhead. Table 1 summarizes the reviewed privacy protection methods in terms of their defensive approaches and compares their performances for the triad of privacy rate, system’s communication latency, and system’s energy consumption.

## 8. Conclusions

Smart homes are IoT-based systems that provide in-home services to residents for their convenience and for control over their domestic lives. However, these technologies have some cyber security flaws that expose them to data leakage, such as wireless snooping-based side-channel attacks. This issue implies the necessity of protecting residents’ personal information by employing a robust privacy-preserving mechanism to prevent the consequences of probable data breach incidents.

A side-channel attack is a sort of cyber attack that exploits the contextual data of cyber–physical systems to infer their functionalities and secret information without gaining access to their source codes or their communication contents.

FATS attacks are effective SCAs that attackers launch on smart homes to eavesdrop on the home’s wireless traffic to obtain the in-home activities passively. Reportedly, the success rate of FATS attacks in detecting and identifying in-home events reaches over 80%.

In this paper, we have reviewed the fundamental strategies to counter FATS attacks and the existing methods in the literature to investigate their strengths and weaknesses, providing novel insight into research gaps and potential opportunities to tackle this imposed threat on smart homes. 

As far as our research has revealed, the principal protection strategies that the existing solutions have been built upon include the temporal manipulation of signal traffic, the injection of fake patterns into the traffic, or a combination of these techniques.

Reviewing the existing protection methods against FATS attacks indicates the following findings:

1. The obfuscation of the home network’s wireless traffic patterns is the predominant approach for confusing the attack algorithm, which diminishes the event detection rate (EDR) and, consequently, the accuracy of the attack. This aim is obtained by manipulating data packet-forwarding processes. Delaying the reporting of events, fake packet injections, or a combination of these are applied techniques for this purpose.
2. Randomness is a key part of the traffic-obscuring techniques that makes it hard for an attack to find the real correlations between the signals sent by the devices.
3. Delay-based methods are not preferred because they hurt the quality of service of the system. This is because the communication latency is too much for delay-sensitive services in the home and it stops them from working effectively.
4. Privacy protection methods impact the energy efficiency of the system. The evidence indicates that to achieve the desired privacy rate, the number of fake injections is usually multiple times more than that of the actual packets; moreover, since the randomness of the dummy packet injections puts the number of transmissions out of one’s control, the system’s energy resources might suffer from a massive overhead caused by the protection technique.
5. The achieved trade-off in the triad of privacy rate, system latency, and energy consumption is a comprehensive metric for evaluating the adequacy of a privacy-preserving method.

Changing the assumptions related to smart home configurations, increasing the number of residents, and improving the capabilities of attacks can lead to further research questions and provide future work opportunities in protecting the privacy of smart building users. However, establishing an optimal trade-off between the system’s critical parameters by maximizing home privacy, compliance with the QoS requirements, and energy demand affordability should be the common focal point of all efforts in the future.

## Figures and Tables

**Table 1:** Comparison of the existing solutions against FATS attacks.

Protection Method	Solution Concept	Performance Metrics		
		Privacy	Latency	Energy
ConstRate [43]	Random fake packets injection in constant intervals	High	Very High	Very High
ProbRate [43]	Random fake packets injection; the intervals shortening exponentially	High	High	Very High
FitProbRate [43]	Random fake packets injection; the intervals shortening exponentially	High	High	Very High
Behavioral semantics analysis [55]	Random fake packets injection, using the nearest exponential intervals for actual transmission	Medium	Medium	High
SDASL [45]	Adaptive fake packets injection	Medium	Medium	High
Actual activity mimicking [56]	Random fake activities injection	High	Low	Medium

## Data Availability

Not applicable.
